# Event-related potentials following contraction of respiratory muscles in pre-term and full-term infants

**DOI:** 10.1016/j.clinph.2019.09.008

**Published:** 2019-12

**Authors:** Kimberley Whitehead, Laura Jones, Maria Pureza Laudiano-Dray, Judith Meek, Lorenzo Fabrizi

**Affiliations:** a Department of Neuroscience, Physiology and Pharmacology, University College London, London WC1E 6BT, United Kingdom; b Elizabeth Garrett Anderson Obstetric Wing, University College London Hospitals, London WC1E 6DB, United Kingdom

**Keywords:** Somatosensory, Proprioceptive, Afferent, Diaphragm, Hiccup, Evoked potential

## Abstract

**Objective:**

Involuntary isolated body movements are prominent in pre-term and full-term infants. Proprioceptive and tactile afferent feedback following limb muscle contractions is associated with somatotopic EEG responses. Involuntary contractions of respiratory muscles, primarily the diaphragm – hiccups – are also frequent throughout the human perinatal period during active behavioural states. Here we tested whether diaphragm contraction provides afferent input to the developing brain, as following limb muscle contraction.

**Methods:**

In 13 infants on the neonatal ward (30–42 weeks corrected gestational age), we analysed EEG activity (18-electrode recordings in six subjects; 17-electrode recordings in five subjects; 16-electrode recordings in two subjects), time-locked to diaphragm contractions (n = 1316) recorded with a movement transducer affixed to the trunk.

**Results:**

All bouts of hiccups occurred during wakefulness or active sleep. Each diaphragm contraction evoked two initial event-related potentials with negativity predominantly across the central region, and a third event-related potential with positivity maximal across the central region.

**Conclusions:**

Involuntary contraction of the diaphragm can be encoded by the brain from as early as ten weeks prior to the average time of birth.

**Significance:**

Hiccups – frequently observed in neonates – can provide afferent input to developing sensory cortices in pre-term and full-term infants.

## Highlights

• Diaphragm contraction in neonates evoked a sequence of three event-related potentials.
• Hiccups can be encoded by the brain as early as ten weeks prior to average time of birth.
• Hiccups – frequent in neonates – provide afferent input to the developing brain.

## 1 Introduction

Involuntary isolated limb movements are prominent in pre-term and full-term infants (Fukumoto et al., 1981, Hadders-Algra et al., 1993, Grigg-Damberger, 2016). Unilateral hand movements evoke somatotopic electroencephalography (EEG) activity overlying the contralateral fronto-central scalp area (Milh et al., 2007, Whitehead et al., 2018b), while bilateral body movements evoke symmetrical EEG activity (Losito et al. 2017). This coupling indicates that frequent limb muscle contractions in the perinatal period may provide afferent input to the developing cortex, which in neonatal animal models allows the refinement of body surface representations (Khazipov et al., 2004, Tiriac et al., 2012).

Alongside the external body map, the mature somatomotor cortex has dedicated areas representing the internal body environment, including the thoracic cavity (Maskill et al. 1991). These are crucial for monitoring the status of vital functions such as breathing, and thereby allow adaptive motor control of respiratory musculature (McKay et al., 2003, Wheeler-Hegland et al., 2011). As for external body representations, these *internal* body maps may also require early afferent input for their development.

In parallel with the high frequency of involuntary limb muscle contractions, involuntary contractions of respiratory muscles – hiccups – are also common in the equivalent of the last trimester of gestation: they are a dominant motor activity pattern in pre-term infants, occupying an estimated 1% of the day (Lipton et al., 1964, Swann, 1978, Brouillette et al., 1980, van Woerden et al., 1989, Pillai and James, 1990, de Vries and Fong, 2006). Hiccups typically occur in bouts, which last for approximately 8 minutes, and happen predominantly during active behavioural states in fetuses, and during wakefulness in neonates (Wagner, 1939, Brouillette et al., 1980, Pillai and James, 1990). These events are a form of reflex motor activity. The efferent limb is mainly the phrenic and external intercostal nerves, which trigger contraction of the diaphragm primarily and the intercostal muscles, as well as the vagus nerve which innervates the striated muscles of larynx and pharynx (Video 1) (Kahrilas and Shi, 1997, Kandel et al., 2000). The afferent limb of the hiccup reflex arc is poorly defined but appears to be mediated by multiple tracts including the phrenic and vagus nerves (Kahrilas and Shi, 1997, Ceriani et al., 2010). To investigate whether these respiratory muscle contractions could provide afferent input to the developing cortex from the internal body environment, we analysed EEG time-locked to hiccups in pre-term and full-term neonates.

## 2 Methods

### 2.1 Subjects

**Supplementary figure 1:**
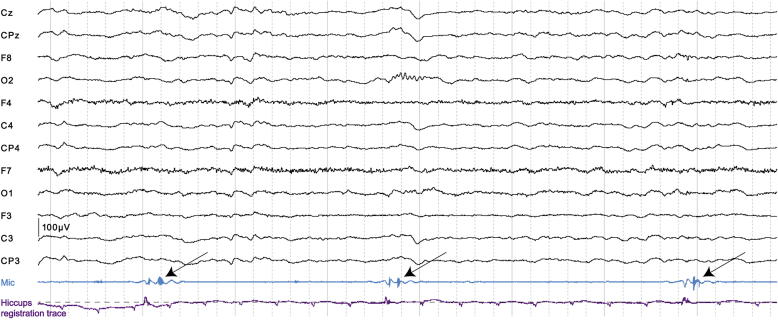
Examples of the stereotyped sound time-locked to each hiccup event. EEG and audio recordings (Mic: signal proportional to sound intensity as recorded with a microphone affixed to the collar of subject #6’s clothing (Unimed electrode supplies)) in which three hiccups occur. Note the stereotyped sound associated with abrupt closure of the glottis (arrows) following the hiccup event onset identified by thresholding (dashed horizontal line) the signal of the hiccups registration trace (in this infant, ECG recording from upper trunk). EEG is displayed referred to Fz (acquisition montage) and with a bandpass filter of 1.5-70 Hz. Scale bar bottom left. Solid grey vertical lines mark each second and dashed grey vertical lines mark each 200 milliseconds.

We identified infants who had hiccups by reviewing 217 research EEG recordings, each from a unique subject with corrected gestational age at study (CGA) 28 + 2–47 + 6 weeks + days, acquired between September 2015 and March 2019 (the CGA and other demographic details of the subset of infants who had hiccups are presented in Table 1). Infants who required mechanical ventilation were unsuitable due to difficulty in accessing EEG electrode placement sites, but all other infants including those who required a low to moderate degree of respiratory support (High flow oxygen or Continuous Positive Airway Pressure) were eligible. Research complied with the Code of Ethics of the World Medical Association (Declaration of Helsinki) and ethical approval was obtained from the NHS Health Research Authority. Parents gave informed written consent, and separate informed written consent was obtained to publish video of one infant. EEG was recorded for approximately 70–90 minutes, in line with recommended best practice (Shellhaas et al. 2011). The presence of a bout of hiccups was recorded at the cot side, alongside annotations of the infant’s vigilance state which was categorised according to behavioural, respiratory and EEG criteria: wakefulness and active sleep are both characterised by movement, irregular breathing, and largely continuous relatively low voltage EEG (Supplementary Fig. 1); quiet sleep is characterised by the absence of movement, regular breathing, and an EEG pattern which fluctuates in amplitude (Tsuchida et al., 2013, Grigg-Damberger, 2016, Whitehead et al., 2018a).

**Table 1:** Clinical data of infants who had hiccups.

Subject/Sex	CGA	GA+PNA	Neurology	Respiratory support	No. epochs analysed
#1/F	30 + 3	27 + 6 + 18	Normal	High flow oxygen	118
#2/M	30 + 4^a^	27 + 3 + 22	Normal	High flow oxygen	49
#3/M	32 + 6	26 + 4 + 44	Normal	High flow oxygen	80
#4/M	33 + 6	32 + 6 + 7	Mild ventriculomegaly (L > R)	Nil	94
#5/M	34 + 5	33 + 6 + 6	Normal	Nil	82
#6/F	34 + 5^a^	34 + 0 + 5	Normal	Nil	138
#7/F	34 + 6	23 + 5 + 78	GM-IVH (grade III R > L)	High flow oxygen	234
#8/F	35 + 5	35 + 3 + 2	Normal	Nil	7
#9/M	35 + 6	30 + 0 + 41	GM-IVH (IPL R; grade I L)^b^	Nil	42
#10/F	36 + 5^a^	35 + 6 + 6	Normal	Nil	83
#11/F	37 + 1	35 + 3 + 12	Normal	Nil	22
#12/M	37 + 5	37 + 2 + 3	Normal	Nil	132
#13/F	42 + 0	41 + 3 + 4	Normal	Nil	235
**Median**	34 + 6	33 + 6 + 7			

CGA = corrected gestational age at study (weeks + days); GA = gestational age at birth (weeks + days); PNA = postnatal age (days). CGA is defined as gestational age at birth plus postnatal age. For example, an infant born at 35 weeks + 2 days, who is 3 days old, is CGA 35 weeks + 5 days. Term is defined as ≥ 37 weeks.

GM-IVH = germinal matrix-intraventricular haemorrhage; IPL = intraparenchymal lesion secondary to GM-IVH. R = right; L = left.

a These infants were in active sleep at onset of hiccups. All other infants were awake.

^b^ Magnetic resonance imaging (MRI) delimited the intraparenchymal lesion to the right basal ganglia, thalami and posterior limb of the internal capsule.

### 2.2 EEG recordings

Eighteen recording electrodes (disposable Ag/AgCl cup electrodes) were positioned individually by a clinical neurophysiologist (KW) according to the international 10/20 electrode placement system (F7, F8, F3, F4, Cz, C3, C4, T7, T8, P7, P8, O1, O2), with additional central-parietal and temporal coverage (CPz, CP3, CP4, TP9, TP10). In 2/13 infants, 2/18 electrodes were sacrificed because the infant became slightly unsettled during set-up (F7/F8 or TP9/TP10). The EEG reference electrode was placed at Fz. Target impedance was < 10 kΩ (André et al. 2010).

### 2.3 Polygraphy recordings

**Fig. 1:**
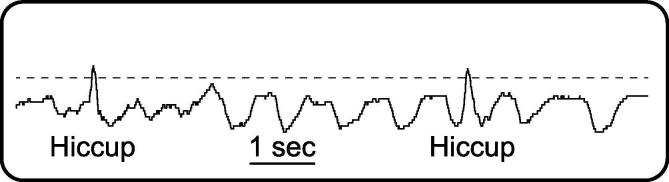
**Representative 10-second long hiccups registration trace (movement recording from lower trunk) in which two hiccups occur, from subject #3.** The event onset (0 ms) is identified by thresholding (dashed horizontal line) this signal.

A movement transducer was applied to the lower trunk and a single lead I ECG was recorded from the upper trunk, both time-locked to the EEG recordings (Video 1). After a bout of hiccups was annotated at the cotside, one of these recordings was utilised offline as a hiccups registration trace (lower trunk 10/13 infants, upper trunk 3/13 infants). Each individual contraction was identified by thresholding this signal, on which a deflection occurred with each event (Fig. 1, Supplementary Fig. 1).

### 2.4 Pre-processing

**Fig. 2:**
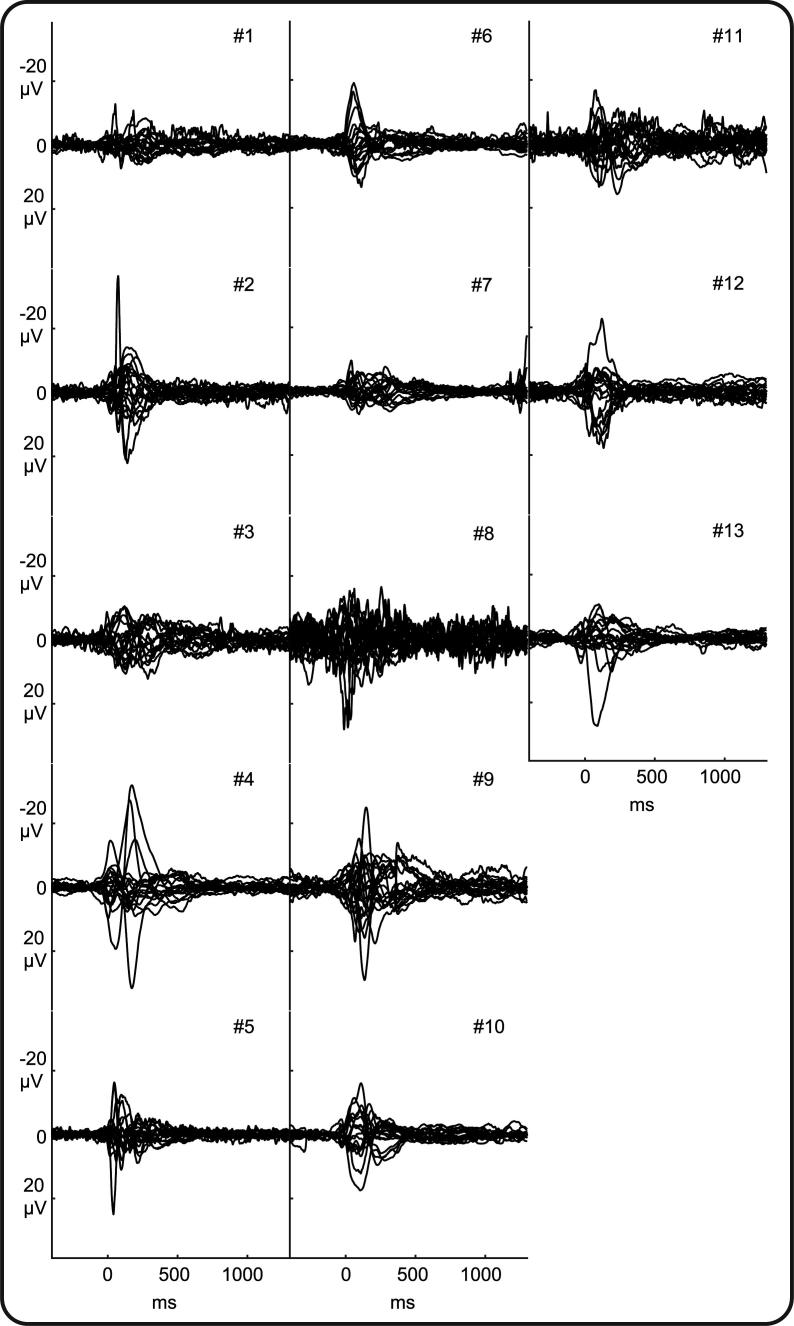
**Individual EEG responses following hiccups.** Butterfly plots of each recording electrode for each of 13 infants (Table 1). Negative amplitudes are plotted upwards as per convention.

Data pre-processing was carried out using Curry v.7, EEGLAB v.14, and custom-written MATLAB code. EEG data were bandpass filtered at 1.5–40 Hz (2nd order Butterworth filter) with a 50 Hz notch filter (4th order Butterworth filter) and then epoched from −400 until  +1300 ms around event onset. One epoch containing movement artefact was discarded from three datasets, and two datasets were de-noised using independent component analysis (component representing ECG breakthrough was removed) (Onton and Makeig 2006). The number of epochs analysed per infant was: 235, 234, 138, 132, 118, 94, 83, 82, 80, 49, 42, 22, and 7 (resulting in 1316 epochs analysed) (Table 1). The median time interval between events was 3.1 seconds (inter-quartile range: 2.1 seconds, minimum interval: 1.2 seconds). The number of epochs analysed per infant was not associated with the median interval between their hiccups (Pearson correlation p = .520, IBM SPSS version 25). Missing and discarded electrode recordings were estimated with spherical interpolation as implemented in EEGLAB v.14 (1/18 electrode recordings were discarded due to artefact in five infants). All EEG epochs were re-referenced to common average (retrieving the reference channel Fz) and baseline corrected by subtracting the mean baseline signal (-400 to 0 ms). Individual responses were estimated by averaging epochs within subject. The signal to noise ratio of each subject’s response was not associated with the number of epochs analysed (Supplementary Data; Fig. 2 visually demonstrates the good signal to noise ratio for each infant independently of number of epochs analysed).

### 2.5 Analysis of hiccup event-related potential

The presence of an event-related potential (ERP) was established using the Topographic Consistency Test, which examines if and at what latencies a stimulus consistently elicits the same scalp field distribution across subjects using Global Field Power (GFP) measurements (standard deviation of the recordings across electrodes at each time point) analysed with non-parametric permutation statistics timepoint by timepoint (n = 1000 randomization runs among channels) (Koenig and Melie-García 2010). Data analysis was implemented in Ragu (Koenig et al. 2011). Statistical significance threshold was set to 0.05 for all tests. An ERP was considered significant if the time period in which the test resulted in p < .05 exceeded 30 ms. Unlike methods to control for multiple comparisons such as false discovery rate, this considers the probability that consecutive samples pass the significance threshold (Guthrie and Buchwald 1991). To provide a visual representation of the topography of each ERP, we generated grand average and individual subject scalp field maps. To facilitate the comparison of topographies across individual subjects, the scalp field map of each ERP for each individual subject was symmetrically scaled to its own peak value.

## 3 Results

Six percent of infants (13/217, Table 1) had a bout of hiccups during their EEG study, with median duration of 7 consecutive minutes (range 1–16). In line with previous reports (Wagner 1939), a bout of hiccups was more likely to occur in infants who were awake during EEG monitoring (chi-squared test (n = 217): p = .005, Phi 0.193; 10 infants were awake at the onset of hiccups, and 3 infants were in active sleep). On the other hand, the incidence of a bout of hiccups was not associated with CGA (binary logistic regression using the Enter Method (n = 217): p = .144).

Cot side observation indicated that hiccups were not associated with changes in respiratory rate (e.g. Fig. 1) or oxygen saturation level (available in 10/13 infants), and statistical analysis demonstrated that the heart-rate of the infants was also unaffected (mean 150 beats per minute (standard deviation (SD): 16) immediately prior to the hiccup bout and 149 beats per minute (SD: 15) immediately after the hiccup bout, LabChart HRV software: paired t-test p = .925 (n = 11 because of poor ECG quality in two infants)). Taken together these data indicate that hiccups were well-tolerated by this cohort, which is in line with previous reports of hiccups in non-mechanically ventilated infants (Brouillette et al., 1980, Niemarkt and Andriessen, 2012).

**Fig. 3:**
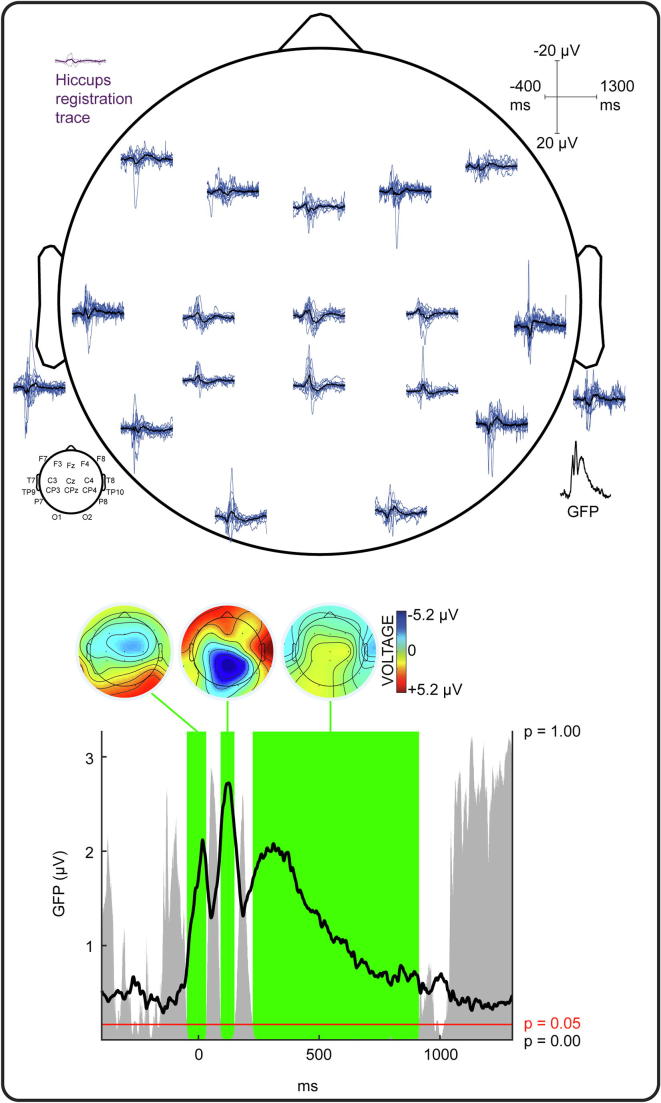
**Grand average of the EEG responses following hiccups.** Upper panel: Hiccups registration trace (purple solid line) and standard deviation (dashed lines); EEG recordings at each electrode from individual infants (blue lines) and grand average (black lines); Global Field Power (GFP) of the grand average EEG recordings. Negative amplitudes are plotted upwards as per convention. Lower panel: GFP of the grand average EEG recordings showing timing and duration of consistent EEG activity across subjects, i.e. event-related potentials (green shading) and their topographies (averaged across their duration as defined by the Topographic Consistency Test). The height of the grey area indicates the p-value of the Topographic Consistency Test. (For interpretation of the references to colour in this figure legend, the reader is referred to the web version of this article.)

**Fig. 4:**
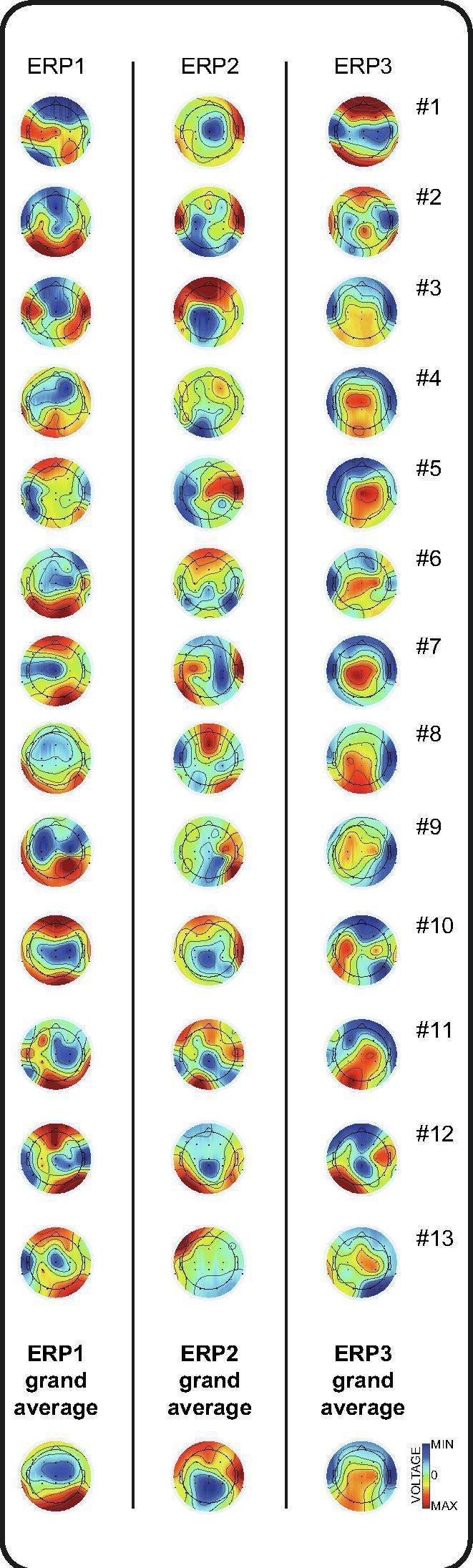
**Individual EEG topographies following hiccups.** Topographies of each event-related potential (averaged across their duration as defined by the Topographic Consistency Test, and individually symmetrically scaled to their own peak value) for each of 13 infants (Table 1) and the grand average. (MIN = Minimum voltage (-µV); MAX = Maximum voltage (+µV)).

**Supplementary figure 2:**
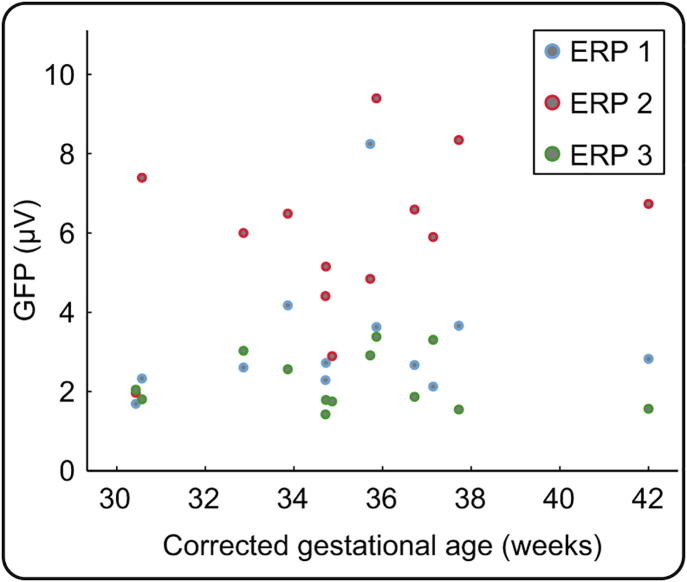
The strength of the EEG responses following hiccups was not associated with corrected gestational age. Dots represent the Global Field Power (GFP) of each individual infant’s EEG recordings during three event-related potentials (ERP 1-3).

Diaphragm contraction evoked a change in EEG activity compared to baseline for every infant (illustrated in Fig. 2). (This included the two infants with germinal matrix-intraventricular haemorrhage (Table 1), in line with reports that sensory responses can be evoked in infants with this injury (Slater et al., 2010, Nevalainen et al., 2015)). Even if there was inter-subject variability, diaphragm contraction-related EEG activity had statistically consistent topography across infants, i.e. ERPs, between −49 to 35 ms (GFP peak latency: 16 ms), 91 to 150 ms (GFP peak latency: 125 ms), and 223 to 913 ms (GFP peak latency: 310 ms) (Fig. 3, Fig. 4). The first ERP comprised fronto-central-temporal negativity, with positivity most prominent across the posterior region. The second ERP comprised central and posterior negativity, with positivity most prominent across the anterior and bi-temporal regions. The third ERP comprised central and posterior positivity, with negativity most prominent bi-temporally (except in the very youngest subject #1 (Fig. 4)). The strength of this event-related EEG activity was not associated with the CGA of the subjects (mean Global Field Power across the latencies the stimulus elicited topographically consistent EEG activity, Pearson correlations: first ERP: p = .561, second ERP: p = .216, third ERP: p = .774; Supplementary Fig. 2).

## 4 Discussion

Diaphragm contraction can evoke a clear cortical response in neonates between 30–42 weeks CGA. This shows that hiccups provide afferent input to the cortex over the equivalent of the last trimester of gestation.

The first two potentials recorded have comparable topography to potentials recorded in neonates up to 185 ms following bilateral myoclonus (Losito et al. 2017) and mechanical somatosensory stimulation of limbs and face, which are somatotopically distributed in pre- and early-term infants (Desmedt and Manil, 1970, Hrbek et al., 1973, Laget et al., 1976, Karniski et al., 1992, Taylor et al., 1996, Pike et al., 1997, Pihko et al., 2004, Tombini et al., 2009, Fabrizi et al., 2011, Nevalainen et al., 2015, Donadio et al., 2018, Whitehead et al., 2019). In older children and adults, perception of respiratory muscle contraction, and other signals from the thoracic cavity, is associated with a sequence of scalp-recorded ERPs across the fronto‐central region lasting until 600 ms post stimulus (Macefield and Gandevia, 1992, Davenport et al., 2000, Frøkjær et al., 2011, Gentsch et al., 2019). In non-human primates, comparable short-latency potentials are recorded from the cortical surface of trunk representation of primary somatosensory cortex, as well as motor and posterior parietal cortex (Amassian 1951). The initial potentials following diaphragm contraction may therefore reflect encoding of afferent input associated with respiratory muscle contraction within the developing somatosensory cortex.

The final potential, positive across the central region, has a similar topography to that recorded in neonates between approximately 200–315 ms following bilateral myoclonus (Losito et al. 2017) and somatosensory stimulation of the body, but is much longer lasting (Karniski et al., 1992, Nevalainen et al., 2015, Whitehead et al., 2019). Consequently, the later part of the cortical response associated with hiccups may differ, at least in part, from that following simple somatosensory feedback. Hiccups are sometimes associated with auditory input produced by abrupt closure of the glottis (Video 1; Supplementary Fig. 1) (Wagner, 1938, Lewis, 1985). Simple auditory stimuli evoke an ERP in neonates with latency and scalp topography very similar to the final potential observed here (Fifer et al., 2010, Chipaux et al., 2013, Kaminska et al., 2018). Therefore, this final potential following diaphragm contraction could encode a *multi*-sensory stimulus. Further, the stimulus here is most often processed while the infant is awake. To our knowledge this is the first study of neonatal cortical responses, of any modality, largely acquired during wakefulness, because newborns spend so little time awake (Curzi-Dascalova et al. 1993). During wakefulness - i.e. a state of heightened attention - sensory information may be encoded differently. For example, in adults long-latency ERPs are only recorded if the stimulus has entered awareness (Libet et al., 1967, Kitazawa, 2002). Although animal models indicate that, until postnatal day 11, afferent input following body movements during wakefulness is relatively unlikely to evoke cortical activity (Tiriac et al., 2014, Dooley and Blumberg, 2018), here we show in human infants 30–42 weeks CGA that brief contractions of a discrete set of respiratory muscles during wakefulness can evoke pronounced cortical responses of similar strength across the age range.

The cortical response characterised in this study is clearly not explained by hiccup-related movement artefact (i.e. electrodes displacement) because a) it is topographically organised, especially across the central region, b) the central electrodes are less likely to be affected by movement as they are placed at the crown of the head and therefore do not brush against bedding or the caregiver (Scher 2006), c) the morphology of the full response recorded does not resemble the movement recorded by the hiccups registration trace and lasts for much longer (Fig. 3).

Fetal ultrasound imaging demonstrates that hiccups are present from just nine weeks gestational age, at which time they are particularly frequent, and then plateau across the third trimester (Pillai and James, 1990, de Vries and Fong, 2006). Therefore repetitive contractions of the diaphragm are one of the earliest established motor activity patterns within the rudimentary functional systems of the fetus. We show here that the sensory feedback from these contractions can be encoded by the brain from as early as 30 weeks CGA, ten weeks prior to the average time of birth. The establishment of early sensory circuits is a crucial developmental milestone for newborn infants (Fabrizi et al. 2011). Our study demonstrates that contractions of respiratory muscles provide sensory input from the internal body environment to the developing brain and may provide the necessary information for the formation of interoceptive representations. This would explain the marked prevalence of hiccups in neonates compared to adults (Brouillette et al. 1980).
