# Supplementary material for: Event-related potentials following contraction of respiratory muscles in pre-term and full-term infants
Source: Clin Neurophysiol. 2019 Dec;130(12):2216–21. doi: 10.1016/j.clinph.2019.09.008 (PMC6907098; doi:10.1016/j.clinph.2019.09.008)
Supplement: Supplementary data 1 [file mmc1.docx]

**Supplementary Results**

Individual responses were estimated using a different number of epochs (between 7 and 235), which may result in differing signal to noise ratios. However, here this variability in the number of epochs analysed did not affect the signal to noise ratio of the individual averages (signal defined as mean Global Field Power across the latencies the stimulus elicited a topographically consistent EEG activation across subjects, i.e. event-related potential; noise defined as mean Global Field Power across the latencies of the baseline period with no topographically consistent EEG activation) (p ≥ .273, Pearson correlations).
